# Chimeric Anterolateral Thigh Flap in Skull Base Reconstruction: A Case-Based Update and Literature Review

**DOI:** 10.3390/brainsci11081076

**Published:** 2021-08-17

**Authors:** Anna Maria Auricchio, Edoardo Mazzucchi, Alessandro Rapisarda, Giovanni Sabatino, Giuseppe Maria Della Pepa, Giuseppe Visconti, Marzia Salgarello, Alessandro Olivi, Giuseppe La Rocca

**Affiliations:** 1 Institute of Neurosurgery, Fondazione Policlinico Universitario A. Gemelli IRCCS, Catholic University, 00168 Rome, Italy; anna.maria90a@libero.it (A.M.A.); alerapi91@gmail.com (A.R.); giovanni.sabatino@materolbia.com (G.S.); gdellapepa@hotmail.com (G.M.D.P.); alessandro.olivi@unicatt.it (A.O.); giuseppe.larocca@materolbia.com (G.L.R.); 2 Department of Neurosurgery, Mater Olbia Hospital, 07026 Olbia, Italy; 3 UOC Chirurgia Plastica, Dipartimento Salute della Donna, del Bambino e di Sanità Pubblica, Fondazione Policlinico Universitario A. Gemelli IRCCS, 00168 Rome, Italy; giuseppe.visconti@policlinicogemelli.it (G.V.); marzia.salgarello@unicatt.it (M.S.)

**Keywords:** skull base reconstruction, chimeric free flap, anterolateral thigh flap, skull defects

## Abstract

Oncologic and traumatic neurosurgery may have to cope with the issue of skull base defects, which are associated with increased risk of meningitis, epidural abscess and cerebro-spinal fluid (CSF) leak. The aim of skull base reconstruction is to repair the dural exposure and to separate the intracranial contents from the nonsterile sino-nasal cavities and extracranial space. Currently, many different surgical techniques have been described, and one of the most performed is the use free flap. In the present paper we performed a case-based update and literature review of the use of chimeric anterolateral thigh free flap harvested from rectus femoris, reporting the case of a 68-year-old man with recurrent spheno-ethmoidalis plane meningioma.

## 1. Introduction

Skull base defects are frequent in oncologic and traumatic neurosurgery. The main goal of reconstructive techniques is to avoid complications such as epidural abscess, meningitis and cerebro-spinal fluid (CSF) leak. Various reconstructive options are available. The entity of soft tissue loss and complexity of defect may influence the versatility and availability of free tissue transfer to optimize reconstruction. Hence, there are several reconstructive choices including local cutaneous flaps, pedicled fasciocutaneous flaps and microsurgical free flaps, such as the anterolateral thigh (ALT), radial forearm (RF), fibula, latissimus dorsi and the fascia lata free flap [1,2,3,4,5,6,7,8,9,10,11]. In the case of large and complex defects, the use of local and pedicled flap could be inapplicable because of limited soft tissue availability and scarcely versatile design. Moreover, repairs of skin defects commonly involve craniofacial district and superficial tissues of the head and the neck [6,12,13]. We report a case of a planum spheno-ethmoidalis reconstruction with chimeric musculocutaneous rectus femoris ALT free flap.

## 2. Case Report

A 68-year-old man came to our attention in September 2017 for epidural empyema after skull base meningioma removal.

**Figure 1:**
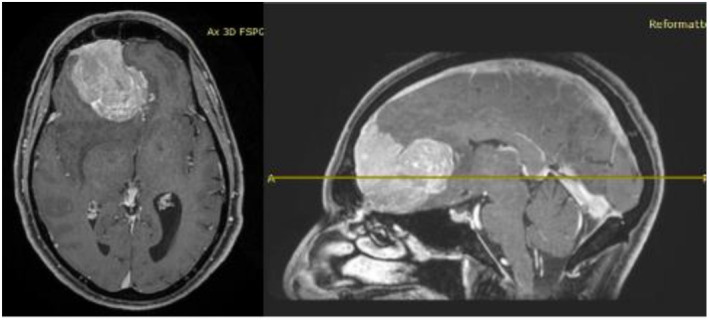
Pre-operative brain MRI showing the anterior cranial fossa meningioma with perilesional oedema.

**Figure 2:**
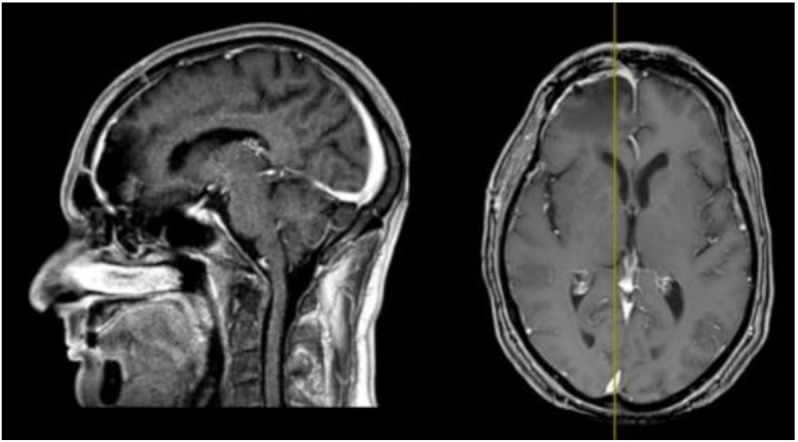
Post-operative brain MRI T1-weighted with contrast images showing the complete removal of the anterior cranial fossa meningioma.

His clinical history started in 2012 with headaches, visual disturbances and behavior’s changes. MRI documented a spheno-ethmoidal lesion with a homogeneous contrast enhancement and with involvement of right anterior skull base (Figure 1). The patient underwent right subfrontal craniotomy with tumor resection. A post-operative MRI showed the apparently complete excision of lesion (Figure 2). Histopathology confirmed the radiological suspicion of meningioma (WHO I). The patient was discharged without complications. 

**Figure 3:**
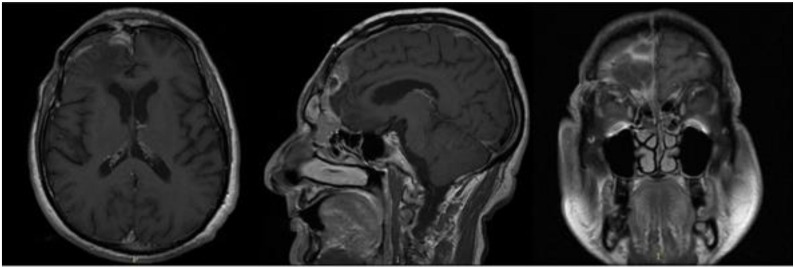
First recurrence of the lesion involving the cribriform plate (T1-weighted with contrast).

In 2016 the mass recurred (Figure 3), and a subtotal resection was obtained with removal of superior and middle nasal turbinates and spheno-ethmoidalis planum that was replaced with a titanium mesh. Histopathological exam revealed an atypical meningioma (grade II, WHO 2016). The patient then underwent hypofractionated radiotherapy (30 Gy) for the residual tumor. 

**Figure 4:**
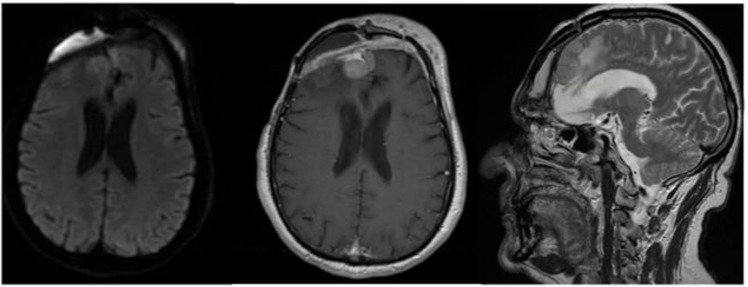
Brain MRI with evidence of a right frontal epidural empyema and meningioma recurrence (left, DWI; center, T1-weighted with contrast; right, T2-weighted).

In September 2017, the patient came to our attention with frontal headache, pyorrhea, right eye swelling, fever and general weakness. The MRI documented the recurrence of the tumor and an evident epidural empyema (Figure 4). We performed tumor resection and revision of the surgical cavity; the titanium mesh was replaced with autologous fascia lata. Frontal operculum was definitively removed. 

**Figure 5:**
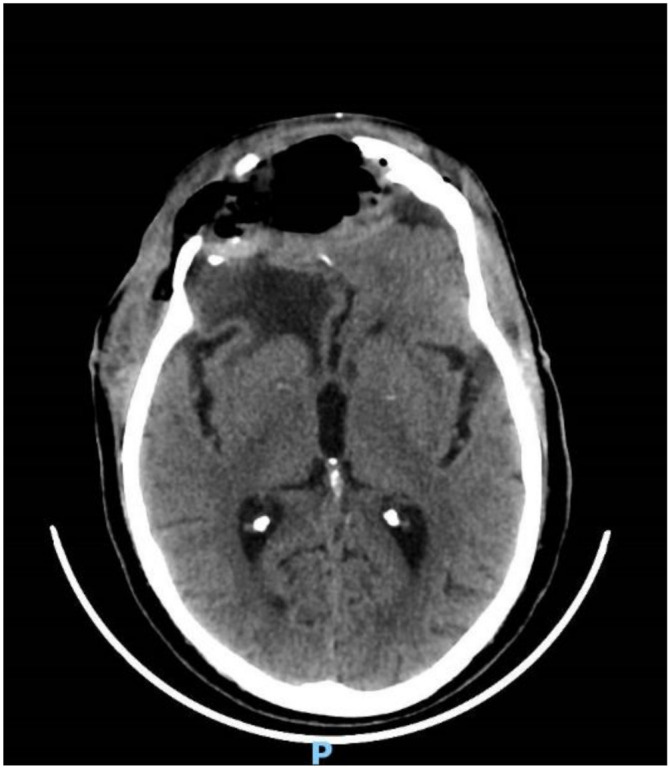
CT head scan showing evacuation of the empyema and post-operative pneumocephalus.

**Figure 6:**
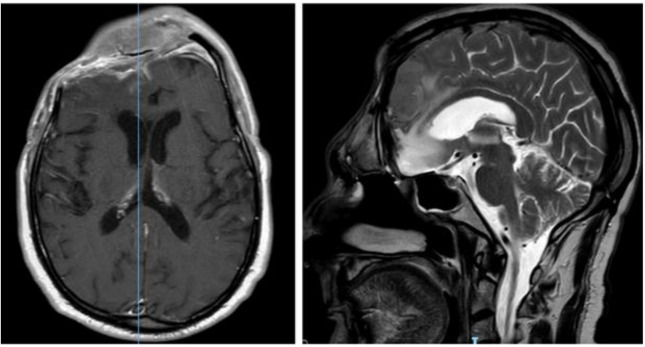
Post-operative brain MRI after evacuation of the epidural empyema, cranial bone flap removal and reconstruction by means of anterolateral thigh flap.

**Figure 7:**
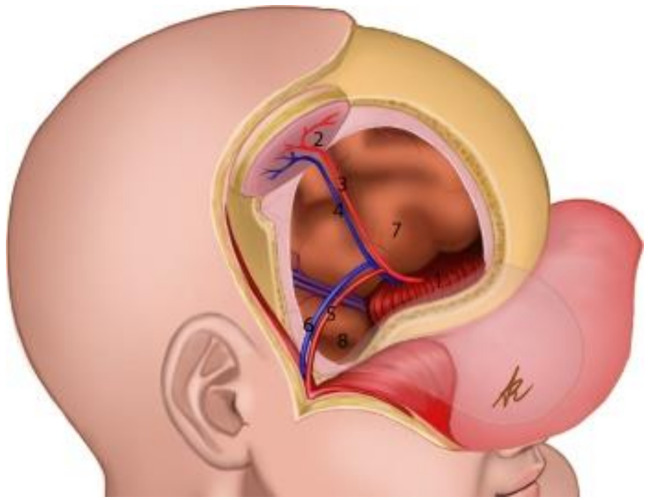
Schematic representation of the reconstruction. (1): Rectus femoris graft reconstructing the anterior cranial fossa; (2): adipocutaneous thigh paddle as the “skin island” of the chimeric flap; (3,4): arterial db-LCFA musculocutaneous (vastus lateralis muscle) perforator of the chimeric flap; (5,6): parietal branch of Superficial Temporal Artery (STA) and its comitantes veins; (7): frontal lobe; (8): temporal lobe.

The CT scan showed removal of tumor and empyema and the appearance of pneumocephalus (Figure 5). A few days later and after antibiotic therapy, we performed a combined right frontal craniotomic and transsphenoidal endoscopic rescue procedure with fascia lata to seal off the cranial base from nose cavity. The post-operative CT scan showed the persistence of pneumocephalus with mass effect. Six days later, we performed a new intervention in collaboration with plastic surgeons: a chimeric perforator anterolateral thigh (ALT) free flap including a superficial monitor skin paddle and a portion of rectus femoris muscle was used to close the spheno-ethmoidalis defect (Figure 6 and Figure 7). The post-operative MRI showed the resolution of the pneumocephalus, and the patient was discharged with no neurological symptoms.

## 3. Surgical Technique

Neurosurgical team reopened the bicoronal incision and removed the fascia lata graft in order to prepare the recipient site. Parietal branches of superficial temporal vessels were dissected free to be used as recipients for microvascular end-to-end arterial anastomosis and venous anastomosis, taking care to spare the frontal branches in order to avoid the risk of devascularization of the frontal radiated scalp flap. The plastic surgeon contemporarily performed an incision along the lateral portion of rectus femoris muscle, and the descending branch of the lateral circumflex femoral artery (db-LCFA) perforators was explored in the suprafascial plane. In order to avoid donor-site complication due to previous fascia lata graft harvest, two muscular branches of db-LCFA distally and supplying the middle third of rectus femoris muscle were traced. The adipocutaneous flap was harvested with a 0.7 mm db-LCFA musculocutaneous perforator, 1 cm laterally to the explorative incision and downstream to two muscular branches for rectus femoris: a 5–12 cm rectus femoris muscle chimeric component was harvested. 

The pedicle was placed on right frontal bone and dura, and the muscle chimeric component was used to fill the nasocranial communication by means of bone and dural anchoring stiches. The monitor skin island was located at the right emicoronal suture, allowing a tension-free scalp closure and reducing compression on the flap pedicle. Between superficial temporal vessels and vessels of the flap pedicle, we found only a minor discrepancy of caliber, which was not a significant hindrance for micro-anastomosis. The skin paddle size was 4–8 cm, and the pedicle length after the muscle branch to the rectus femoris muscle was 13 cm. The donor site was then closed primarily without undermining. The reconstruction with the chimeric ALT perforator flap with skin paddle and muscle component allowed the separation of the anterior skull base and the nasal fossa with muscle, using the skin paddle as an external on sight area for monitoring the vitality of the graft.

**Figure 8:**
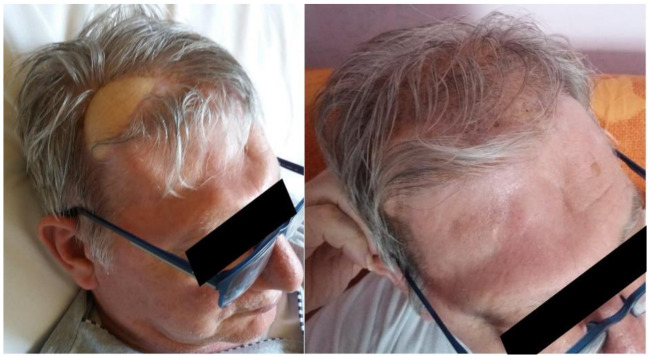
Facial appearance of the patient after surgery. The skin paddle is still recognizable, although easily covered by the hair of the patient.

The patient was discharged without new complications. No functional limitation nor significant discomfort was related to sacrifice of rectus femoris muscle. The resolution of the pneumocephalus was confirmed by post-operative MRI (Figure 6). The patient was satisfied with the functional outcome, and he accepted the aesthetical facial appearance (Figure 8).

## 4. Discussion and Literature Review

Skull base reconstruction after demolitive surgery may be the most delicate phase of the surgical workflow. Many technical options are available. We found chimeric ALT free flap a good solution for a very complicated case of recurrent meningioma. Here, we provide a summary of strengths and weaknesses of this technique to help neurosurgeons that will have to cope with similar clinical situation. 

A chimeric flap consists of multiple otherwise independent flaps that each have an independent vascular supply, with all pedicles linked to a common source vessel [14]. In our case, the chimeric ALT free flap chosen was based on the db-LCFA musculocutaneous perforator with a portion of the rectus femoris muscle flap receiving vascular supply from two muscle branches of the db-LCFA. A CT angiography of the lower limb was not performed before this surgical intervention. This radiological exam could be useful for anticipating the vascular anatomy of the donor site. The vastus lateralis muscle was compromised for previous surgeries with fascia lata harvesting. Moreover, scars in both legs represented a source of complexity in obtaining a vital flap; the same difficulty was found in dissecting the reopened surgical cavity and in performing effective anastomosis. However, the result was aesthetically and functionally accepted by the patient. The skin paddle allowed the surgeon to monitor flap viability and reduce tension at closure. 

The chimeric ALT free flap is a strategical solution with low risk of necrosis, infection and flap failure [15] in delicate cases of injured skin with circumstances where other type of flaps can fail: history of infection, radiation therapy, multiple prior surgeries or loss of soft tissue and bone.

In 1984, Song described the ALT flap, more appropriately named lateral femoral circumflex artery perforator flap, as the more applicable flap for head and neck reconstruction [16,17]. Usually the pedicled flap and other free flaps such as the free omentum flap with skin graft [18], groin flap, latissimus dorsi muscle or musculocutaneous flap/rib perforator [19], radial forearm flap [8,10] and rectus abdominis flap are suitable for small and medium skull defects [20]. However, there are some examples of chimeric ALT free flap of vastus lateralis, such as in craniofacial and plastic surgery for huge maxillary defects, for the reconstruction of oral cavity and neck [6,12,13,21,22,23]. Zaretski in 2006 [24] described a case series of neck and head defects reconstruction with a chimeric ALT, without mentioning the use of this technique in skull base defects. The classification of chimeric ALT free flap considers different femoral anterolateral double island flaps divided into three types: trunk type (type I), in which the perforators of two flaps originated in the descending branch and the transverse branch of the lateral femoral circumflex artery; branch type (type II), in which both the perforators originated in the descending branch or the transverse branch of the lateral femoral circumflex artery; and bifurcation type (type III), in which two perforators originated in the bifurcation of one perforator that originated in the descending branch or the transverse branch of the lateral femoral circumflex artery [17]. Furthermore, concerning the proper neurocranial defects, a good differentiation of regions to be reconstructed can predict the complexity and the risks of postprocedural complications. Herein, most authors classify skull base defects on which of the three cranial fossae are involved [25,26], distinguishing the defects for the anterior (Region I), middle (Region II) and posterior (Region III) fossa. In oncological neurosurgery, when less invasive approaches are not possible [27], the most common flaps used in skull base reconstruction are pedicled ones, while the pericranial and temporoparietal fascia flaps are commonly applied as dural reinforcements [2,28]. Some authors proposed a reconstruction of composite defect of bone and soft tissues with a combined latissimus dorsi and serratus anterior and rib free flap [8,19]. Recent data demonstrate high complication rates for regional pedicled flaps such as the pectoralis major, trapezius and latissimus dorsi muscle and myocutaneous flaps [8,10,29]. Hence, they are infrequently used, although they may still be necessary in patients who are not good candidates for a free flap procedure [2]. The microvascular free flap is mostly applied for large areas of defects since it guarantees more abundant skin substitute than local and regional flap [30]. Hence, the ALT flap has an added advantage of including the fascia lata as a robust and vascularized dural replacement that is effective in preventing leakage of cerebrospinal fluid [31]. Present literature reports few similar neurosurgical cases (Table 1). Cherubino et al. [32] analyzed seven cases with an ALT reconstruction by septocutaneous flap in two cases and musculocutaneous flap in five cases. Only one case of flap failure was described with venous congestion and complete necrosis. Parkes et al. [33] described orbital lesions involving either anterior skull base and maxillofacial district. We only extrapolated orbital lesions and considered the chimeric ALT flap and rectus femoris flap, observing a very low rate of complications. Lo et al. [34] discussed the case of ALT free flap reconstruction with a de-epithelialized skin paddle in a patients affected from meningioma, with no atrophy of tissue at ten months of survival. Posch et al. [35] report 11 cases, 4 of which were skull base tumors. The rate of complication was very low, with a hematoma and an artery occlusion that occurred within three hours from surgery, with uneventful outcome. Hanasono et al. [30] was one of the first authors describing ALT free flap reconstruction in the head and neck district. We selected 31 cases of neurocranial regions and found some complications such as CSF leak, infection and wound dehiscence and seroma. Yano et al. [36] described three cases treated with chimeric ALT flap without complications. On the other hand, Zhao et al. [37] documented the flap failure in a traumatic scalp injury 6 weeks after surgery and performed a rescue procedure based on retrograde blood flow of contralateral superficial temporal artery. Park et al. [38] considered reconstruction of the cranial profile after traumatic injury with a chimeric ALT flap with no complications by using facial artery and vein in anastomosis. Llorente et al. [39] described the use of different free flaps in skull base, including the chimeric ALT flap; the rate of complication for each type of flap and which kind of defect was repaired with chimeric ALT flap was not reported.

*Pros*

- High rate of good results, with relatively low risk of complications as necrosis and infection;
- Adaptable to large cranial defects;
- Additional skin is not necessary;
- Adequate thickness;
- Good microvascular anastomosis;
- Low risk of donor site morbidity [5,15,40,41];
- The well-vascularized fascia components of ALT flaps can be used to seal dural defects and avoid refractory infections in the donor site;
- The plasticity of the flap: it can be harvested as thinned skin and as a fasciocutaneous, myocutaneous or chimeric flap in order to provide the necessary volume in restoring the natural scalp edge [24].

*Cons*

- The difficulty in harvesting represents the most important limitation in its choice [4].

## 5. Conclusions

The chimeric anterolateral thigh flap could be a valuable option in the case of skull base reconstruction. It is effective, safe and aesthetically acceptable for the patient.

## Figures and Tables

**Table 1:** Literature review of chimeric ALT free flap in neurosurgical procedures.

Author	Type of Chimeric ALT Flap (Number of Cases)	Cause of Cranial Defect (Number of Cases)	Neurocranial Defect (Number of Cases)	Complication (Number of Cases)
Present case	Rectus femoris and fascia lata (1)	Atypical meningioma (1)	Sphenoidal planum (1)	-
Cherubino et al. (2017) [32]	Vastus lateralis and rectus femoris (7), with septocutaneous vessels (2) and muscolocutaneous vessels (5)	Squamocellar Carcinoma (2) Adenocarcinoma (1) Melanoma (3) Neuroblastoma (1)	Ethmoid (3) sphenoid (1) frontal sinus (1) orbit (2)	Venous congestion and failure (1)
Parkes et al. (2011) [33]	Vastus lateralis and rectus femoris (1)	Meningioma (4) Squamocellar carcinoma (12) Basal Cell carcinoma (6) Esthesioneuroblastoma (1) Frontal bone osteomyelitis (1) Hemangiopericytoma (1) Basosquamous carcinoma (1) Melanoma (3)	Orbital (29)	Wound infection (2) Hematoma (1) CSF leak (3) Failure (3)
Lo et al. (2011) [34]	Vastus lateralis and de-epithelialized skin paddle (1)	Meningioma (1)	Anterior skull base (1)	-
Posch et al. (2005) [35]	Partial Vastus lateralis (4)	Squamous cell carcinoma (2) Basal cell carcinoma (2)	Parietal frontal orbital (4)	Hematoma (1) Arterial occlusion (1)
	Vastus lateralis (31)	Squamous cell carcinoma (19) Sarcoma (5) Basal cell carcinoma (5) Sebaceous cell carcinoma (1) Acinic cell carcinoma (1)	Anterior fossa (2) Middle fossa (5) Posterior fossa (17) Middle posterior fossa (4) Anterior middle fossa (6)	CSF leakage (3) Infection (3) Wound dehiscence (2) Sieroma (2)
Yano et al. (2016) [36]	Vastus lateralis (3)	Olfactory neuroblastoma (1) Hemangiopericytoma (1) Meningioma (1)	Anterior skull base (2) Middle skull base (1)	-
Zhao et al. (2016) [37]	Vastus lateralis (1)	Trauma (1)	Frontal (1)	Atrophy and failure (1)
Park et al. (2012) [38]	Vastus lateralis and latissimus dorsi (1)	Trauma (1)	Fronto-parietal (1)	-
